# Scientific Items

**Published:** 1884-04-20

**Authors:** 


### SCIENTIFIC ITEMS.

**Electro-generative Torch or Candle.**—Dr. Brand, of La Rochelle, France, has an electro-generative torch or candle, which yields a current of electricity in the act of burning. It is prepared by making a paste of coal dust and molasses and molding it into a stick, which serves as the inflammable wick of a candle. This rod is then covered with asbestos in a thin sheet, and dipped into fused nitrate of potash until a good thick coating of the nitrate adheres. The wick being ignited, it burns away and a current of electricity is drawn from the candle by wires inserted into the nitrate and the coaly wick. Though this current is comparatively feeble, and not as yet of much practical value, the discovery is important as showing the possibility of electro-generative fuels. —*Home Health.*

**A Pocket Steam Heater.**—Connecticut has been the birthplace of many novel inventions, but the device which the New Haven News describes as having been recently produced by a Bridgeport professor is surpassed by none of them in originality and daring. The paper referred to says of this invention, called the "portable body steam heater," that the apparatus is a small affair, consisting of a copper boiler, under which is a diminutive lamp, all incased in a nickel box, and balanced something like a compass, so that, no matter what position the outside box is in, the boiler and lamp will always remain in the required vertical position. The entire apparatus is so small that it can be carried in the pocket. After the lamp is lighted, the water in the boiler is heated and circulated through rubber tubes, which run down the legs, around the ankles, up around the back, and back to the boiler. The circulation of the warm water keeps the body warm on the coldest day. A safety-valve and escape for a higher pressure of steam than the affair is allowed to carry flows off at the back of the wearer's neck. Elaborate heaters are being constructed for ladies' wear. They can be worn inside the bustle, and entirely obscured. Before going out of the house the lady's maid can light the lamp, which, by the way, is gaged to run six, eight or ten hours, and "my lady" walks out under a full pressure of steam, and warranted to keep warm during the promenade.—*Mechanical News.*

**Electric Headlights.**—The Wooley Locomotive Headlight Company have had perfected an invention for lighting headlights by electricity, the machine furnishing power therefor being placed on the engine opposite the air brake. The company have gone largely into the manufacture of electric light for cars, bridges, stations and tunnels. [At the rate the use of this invention increases, it seems that it will shortly be numbered among our household necessities.—*Ed.*]

**New Process for Making Dynamite.**—Although Dynamite is less dangerous to handle than nitro-glycerine, its preparation, involving, as it does, the nitration of the glycerine, is not less easy, nor is it safer. When the glycerine enters the mixture of nitric and sulphuric acid, the reaction takes place with such violence as to produce a large amount of heat.

According to the Polytechnisches Notizblatt, a much safer method is now in use by Boutmy & Faucher in their powder works. Here the operation of nitrating takes place in two stages. The glycerine is first converted into a sulpho-acid by the action of strong sulphuric acid. An acid mixture is prepared separately from equal parts of nitric and sulphuric acid. The two liquids are then mixed. The sulpho-nitric acid absorbs nearly as much heat in liberating the glycerine from its combination as is formed by its combining with the glycerine; hence, it acts as a refrigerant to keep it cool, and the process is not attended with much increase of temperature.

In the new process the nitro-glycerine is not formed so suddenly as in the old way, but slowly and steadily, settles rapidly and can be easily washed, while the yield, it is claimed, is greater. Nevertheless, Boutmy & Faucher have taken every precaution to avoid explosions and to protect the workmen from fumes.—*Druggist's Circular.*

**Electricity in the Slaughter House.**—Mr. George L. Fox, of London, has devised an apparatus for slaughtering animals by electricity. We find the following description of it in an exchange: The animal to be killed has first the top of its head and its feet and its legs wetted with salt water. It is then led into a stall, and made to stand upon an iron plate connected with the negative pole of a condenser of a capacity of about one hundred microfarads. The operator then touches the animal's head with the positive pole, and it falls dead. The death is believed to be painless, because physiologists have demonstrated that a nervous vibration cannot be communicated to the brain in less than the tenth of a second, and another tenth of a second must elapse before a sensation can result from the vibration thus communicated. One-fifth of a second must, therefore, necessarily intervene between the actual infliction of an injury and the experience of pain from it; but the flash from such an apparatus as has been described, presumably paralyzes every fragment of a nerve in man or beast in something like a hundred thousandth part of a second. It is said that, for about two thousand five hundred dollars, such an electrical apparatus as is here alluded to may be fitted up in any stable or outhouse, and that it will kill as rapidly as animals can be placed in position and taken away again.—*Pop. Science News.*

**Rainfall in New Mexico and California.**—Statisticians say the smallest annual rainfall in this country is in New Mexico and California, 13 and 18 inches each; the highest in Oregon and Alabama, 49 and 56 inches.—*Ex.*

